# Secreted frizzled related proteins inhibit fibrosis *in vitro* but appear redundant *in vivo*

**DOI:** 10.1186/1755-1536-7-14

**Published:** 2014-10-02

**Authors:** Ellen De Langhe, Carolina Aznar-Lopez, Vanessa De Vooght, Jeroen A Vanoirbeek, Frank P Luyten, Rik JU Lories

**Affiliations:** 1 Department of Development and Regeneration, Laboratory of Tissue Homeostasis and Disease, Skeletal Biology and Engineering Research Center, KU Leuven, Leuven, Belgium; 2 Division of Rheumatology, University Hospitals Leuven, Leuven, Belgium; 3 Department of Public Health, Experimental Toxicology Unit, KU Leuven, Leuven, Belgium

**Keywords:** Transforming growth factor-beta, WNT signaling, Secreted frizzled related proteins, Bleomycin, Pulmonary fibrosis, Skin fibrosis

## Abstract

**Background:**

The pathogenesis of pulmonary fibrosis remains poorly understood. The Wnt signaling pathway regulates fibrogenesis in different organs. Here, we studied the role of two extracellular Wnt antagonists, secreted frizzled-related protein-1 (SFRP1) and frizzled-related protein (FRZB) on lung fibrosis *in vitro* and *in vivo*. For this purpose, we used an alveolar epithelial cell line and a lung fibroblast cell line, and the bleomycin-induced lung fibrosis model, respectively.

**Results:**

During the course of bleomycin-induced lung fibrosis, *Sfrp1* and *Frzb* expression are upregulated. Expression of *Sfrp1* appears much higher than that of *Frzb. In vitro*, recombinant SFRP1, but not FRZB, counteracts the transforming growth factor β_1_ (TGFβ_1_)-induced upregulation of type I collagen expression both in pulmonary epithelial cells and fibroblasts. Both SFRP1 and FRZB inhibit the TGFβ_1_-induced increase of active β-catenin, but do not influence the TGFβ_1_-induced phosphorylation levels of SMAD3, positioning Wnt signaling activity downstream of the active TGFβ signal in lung fibroblasts, but not in alveolar epithelial cells. *In vivo*, *Sfrp1*^*−/−*^ and *Frzb*^*−/−*^ mice showed identical responses to bleomycin in the lung compared to wild-type controls.

**Conclusions:**

Although SFRP1 counteracts the effect of TGFβ_1_ in pulmonary cells *in vitro*; loss of neither SFRP1 nor FRZB alters fibrotic outcomes in the lungs *in vivo*. The lack of *in vivo* effect in the absence of specific SFRPs suggests functional redundancy within this family of Wnt antagonists.

## Background

Interstitial lung disease can occur in association with connective tissue diseases such as systemic sclerosis (SSc), can specifically present as idiopathic pulmonary fibrosis (IPF), or may be caused by exposure to environmental toxins. The pathophysiology of lung fibrosis remains poorly understood and regardless its cause, the disease processes are associated with significant morbidity and mortality [1]. The current concept suggests injury-induced activation of epithelial cells, with deregulated repair causing inappropriate alveolar regeneration and impaired epithelial-mesenchymal crosstalk, resulting in mesenchymal cell activation, migration, and progressive lung fibrosis [2]. Activation of signaling pathways that have critical roles during embryonic development is thought to play a central role in this abnormal repair response. In this context, increasing evidence supports a crucial part for the Wnt (wingless-type like) signaling pathway [3].

Wnt ligands are lipid-modified, cysteine-rich glycoproteins that bind to frizzled (Fz) receptors. The formation of a ligand-receptor complex associated with a co-receptor LRP5 or LRP6 (low-density lipoprotein receptor-related protein) leads to activation of the canonical Wnt cascade with key mediator β-catenin that upon accumulation translocates to the nucleus and regulates gene transcription [4,5]. In addition, Wnts can also activate non-canonical cascades such as the Wnt/JNK (Jun-amino-terminal kinase), Wnt/Ca^2+^, or Wnt/PKA (protein kinase A) pathway [6].

Cumulating evidence supports a role for activation of Wnt signaling in fibrotic lung disease. Recent data have shown that inhibition of GSK3β, an enzyme that inhibits accumulation of ß-catenin, increases collagen release from SSc fibroblasts and aggravates both bleomycin-induced skin fibrosis and skin fibrosis in Tsk1 mice [7], potentially through an endothelin-dependent mechanism [8]. Furthermore, activation of Wnt signaling through downregulation of the Wnt antagonist Dickkopf (Dkk) is required for TGFβ-mediated fibrosis, positioning Wnt signaling downstream of TGFβ in fibroblasts [9]. Increased nuclear β-catenin has been detected in lung tissue from patients with IPF and systemic sclerosis-associated lung fibrosis, compared to healthy controls [10,11]. Different Wnts and Wnt target genes are upregulated in IPF lungs and specifically localized to bronchial and alveolar type II epithelial cells (WNT1, WNT3A) or endothelial and vascular smooth muscle cells (WNT1) [12]. Further evidence for active WNT signaling in lung fibrosis came from microarray analysis of IPF lungs, confirming enrichment of WNT-related genes encompassing ligands (*WNT2*, *WNT5a*), receptors (*FZ7*, *FZ10*), antagonists (*SFRP1*), and target genes (*LEF1*) [13]. Results from different microarray analysis studies all indicate an upregulation of SFRP1 and FRZB in fibrotic lung disease (IPF). In addition, some animal model data are available. *Wisp1* (WNT1-inducible signaling protein 1) is upregulated in bleomycin induced murine lung fibrosis, as well as in human IPF. Neutralization of WISP1 [14] or inhibition of Wnt/β-catenin/CREB (cyclic adenosine monophosphate (cAMP) response element binding protein) binding protein (CBP) signaling attenuates and reverses bleomycin-induced pulmonary fibrosis [15].

Endogenous modulators and antagonists regulate Wnt signaling in the extracellular space and at the level of the receptors. Secreted frizzled-related proteins (SFRPs) bind Wnt ligands in the extracellular space, thereby theoretically preventing ligand-receptor interaction. Frizzled-related protein (FRZB) was the founding member of this family [16-18] and confirmed to bind xWNT8 and antagonize its activity in *Xenopus*. The SFRP family encompasses five members in humans, SFRP1 to SFRP5, with SFRP3 a synonym for FRZB. Sequence comparison and phylogenetic analysis have demonstrated SFRP1, SFRP2, and SFRP5 to form a subgroup that differs from the one formed by FRZB and SFRP4 [19]. Recent evidence supports the view that FRZB and other SFRPs do not merely act as antagonists but have more complex effects on Wnt gradient formation, including the extension of the ligand signaling range [20,21].

In this work, we aimed to further understand the potential role of SFRPs in lung fibrosis. We therefore studied the effect of SFRPs on lung fibrosis in *in vitro* and *in vivo* models, including the effect caused by absence of endogenous SFRP1 and FRZB in the bleomycin-induced lung fibrosis model. We show that both *Sfrp1* and *Frzb* are upregulated during the course of bleomycin-induced lung fibrosis. *In vitro*, SFRP1 reduces the TGFβ_1_-induced upregulation of collagen expression in both pulmonary fibroblasts and alveolar epithelial cells. *In vivo*, the absence of SFRP1 or FRZB does not alter fibrotic outcomes in the lung, suggesting functional redundancy.

## Results

### In bleomycin-induced lung fibrosis, canonical Wnt signaling is active and Sfrp1 and Frzb are upregulated

**Figure 1:**
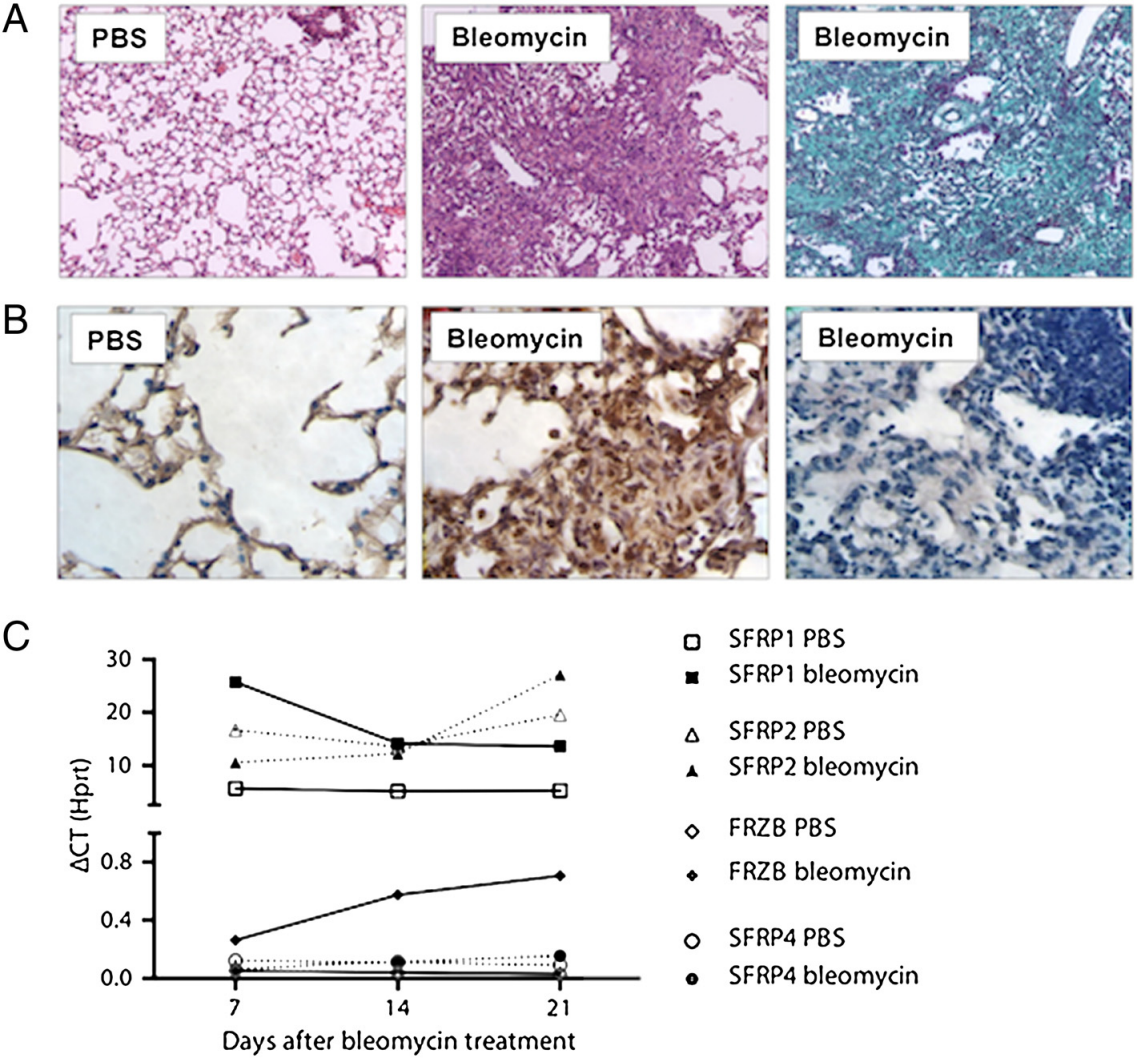
**WNT and SFRP dynamics in bleomycin-induced lung fibrosis. (A)** Representative images lungs from WT mice, 4 weeks after PBS or bleomycin instillation. (Hematoxylin-Eosin and Masson Trichrome staining) **(B)** β–catenin or goat IgG (negative control, 3rd panel) immunohistochemistry, 4 weeks after intratracheal PBS or bleomycin instillation in WT mice. **(C)** Total lung gene expression level of *Sfrp1*, *Sfrp2*, *Frzb* and *Sfrp4* following bleomycin instillation (n = 4, except for bleomycin group at day 21 n = 2; data presented as mean and SEM of ΔCT values normalized to *Hprt* expression).

Intratracheal bleomycin instillation results in pulmonary fibrosis with excessive collagen deposition and obliteration of alveolar structures (Figure 1A). Immunohistochemistry demonstrated increased nuclear β-catenin in fibrotic zones indicating active canonical Wnt signaling, while this signal was absent in the PBS-treated lungs (Figure 1B). We used gene expression analysis of the different *Sfrps* to study their dynamic profile in the bleomycin-induced pulmonary fibrosis model. *Sfrp1* and *Sfrp2* mRNA levels were 2 log-scales more abundant than those of *Frzb* and *Sfrp4. Sfrp5* could not be detected. *Sfrp1* levels were significantly increased at all time points after bleomycin treatment but not different between time points (Figure 1C) (2-way ANOVA *P* = 0.0015 for bleomycin *vs.* PBS, *P* >0.05 for time and interaction). *Frzb* levels were significantly and consistently increased over time after bleomycin treatment (2-way ANOVA *P* <0.0001 for bleomycin *vs.* PBS, *P* = 0.0248 for time and *P* = 0.0154 for interaction). *Sfrp2* and *Sfrp4* levels were not different between groups or during the course of the disease, with *Sfrp2* relative expression similar to *Sfrp1* and *Sfrp4* levels similar to baseline *Frzb*. We therefore further focused on SFRP1 and FRZB.

### SFRP1, but not FRZB, reduces TGFβ_1_-induced collagen upregulation in pulmonary fibroblasts and alveolar epithelial cells

**Figure 2:**
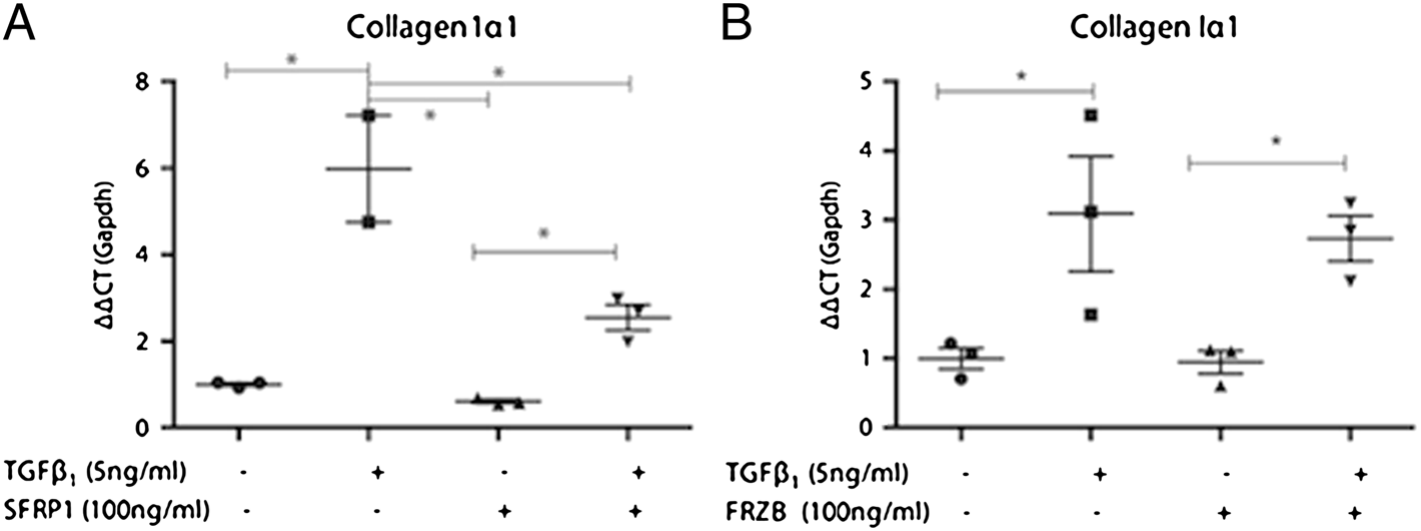
**Effect of SFRP1 and FRZB on pulmonary fibroblasts. (A)** Gene expression level of *collagen1α1* in MRC5 cells, stimulated with TGFβ_1_ and SFRP1; (n = 3; data presented as mean and SEM). **(B)** Gene expression level of *collagen1α1* in MRC5 cells, stimulated with TGFβ_1_ and FRZB (n = 3; data presented as mean and SEM) (*P <0.05 by one-way ANOVA and *post-hoc* test vs TGFβ_1_-only stimulated cells).

**Figure 3:**
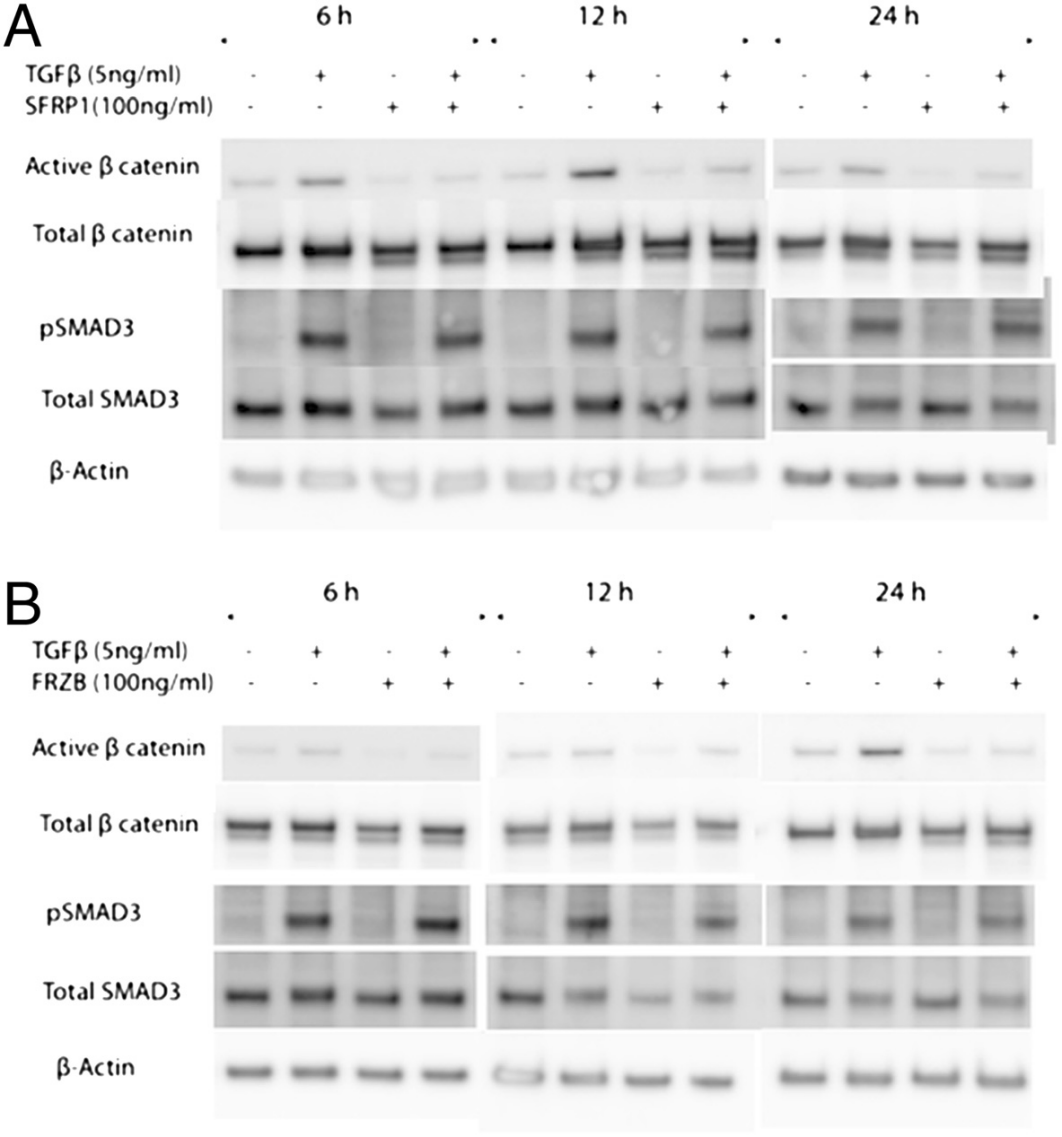
**Downstream signaling in pulmonary fibroblasts after SFRP1 and FRZB stimulation.** Western blot of protein extracts from total MRC5 cell lysates, stimulated with TGFβ_1_ and SFRP1 **(A)** or FRZB **(B)**, labeled with antibodies against pSMAD3, total SMAD3, total β-catenin, active β-catenin, and β-actin.

**Figure 4:**
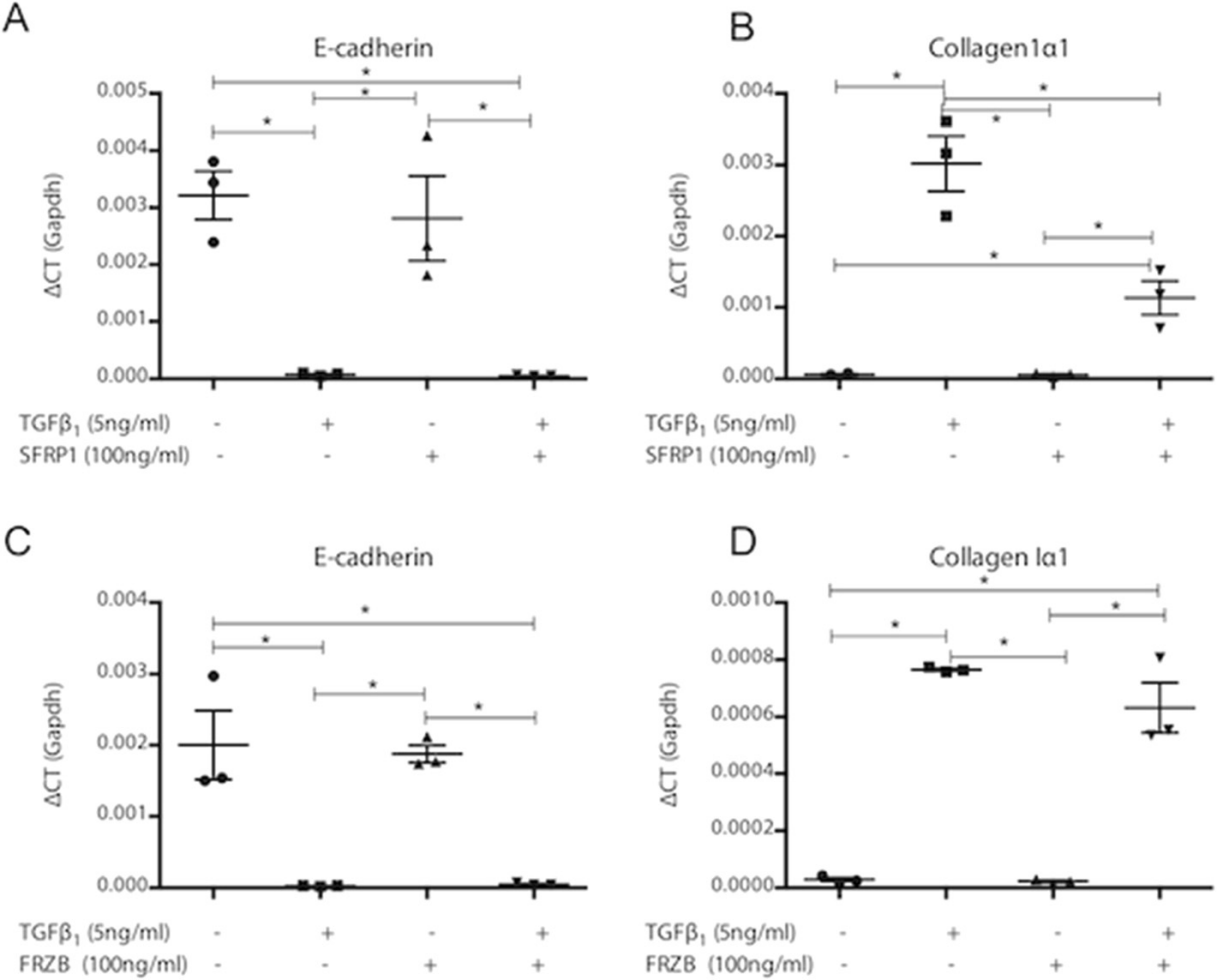
**Effect of SFRP1 and FRZB on alveolar epithelial cells. (A)** Gene expression levels of *E-cadherin* and **(B)***collagen1α1* in A549 cells, stimulated with TGFβ_1_ and SFRP1 (n = 3; data presented as mean and SEM). **(C)** Gene expression levels of *E-cadherin* and **(D)***collagen1α1* in A549 cells, stimulated with TGFβ_1_ and FRZB (n = 3; data presented as mean and SEM) (*P <0.05 by one-way ANOVA and *post-hoc* test *vs.* TGFβ_1_-only stimulated cells).

**Figure 5:**
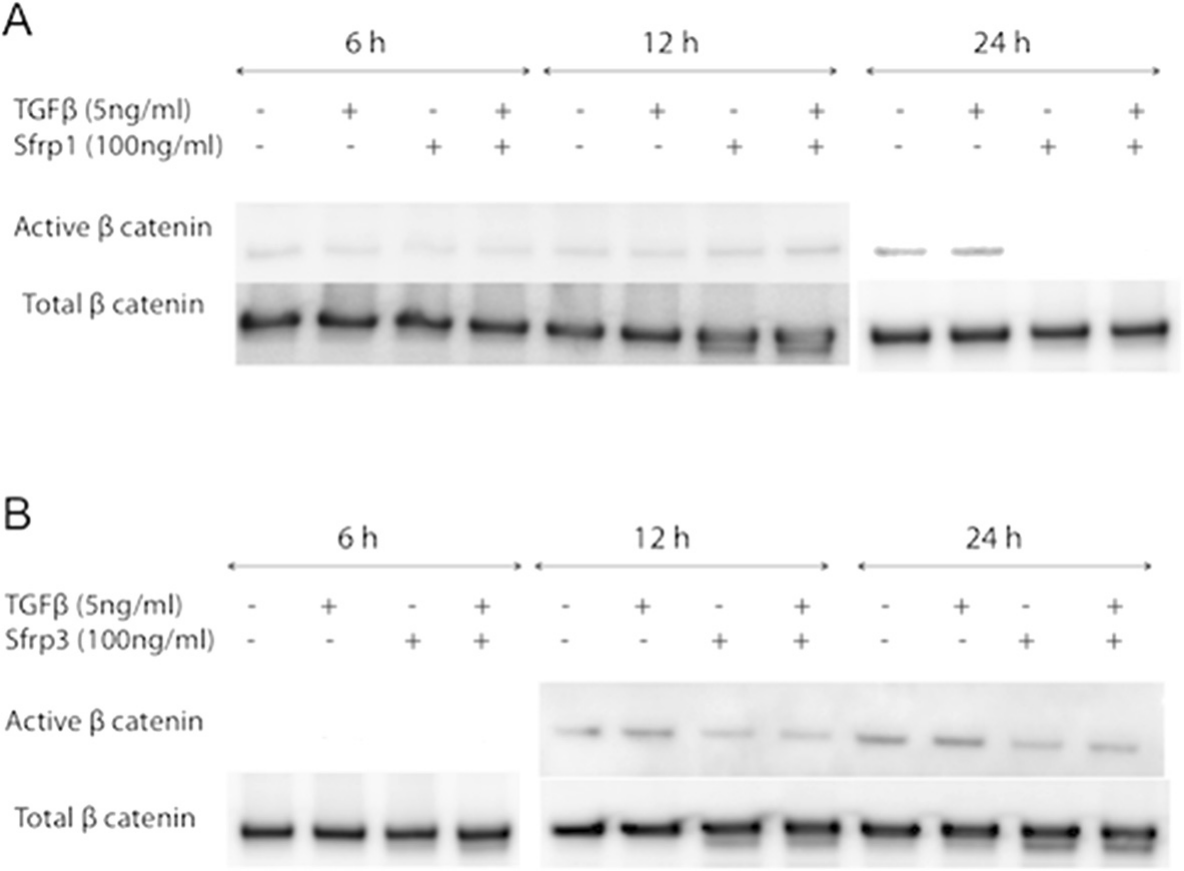
**Downstream signaling in alveolar epithelial cells after SFRP1 and FRZB stimulation.** Western blot of protein extracts from total A549 cell lysates, stimulated with TGFβ_1_ and SFRP1 **(A)** or FRZB **(B)**, labeled with antibodies against total β-catenin and active β-catenin.

Activation of pulmonary fibroblasts is an important process in lung fibrosis. In TGFβ_1_-stimulated fibroblast MRC5 cells, SFRP1 significantly reduced TGFβ_1_-driven *Coll1α1* expression (Figure 2A). In contrast, this effect was absent with FRZB stimulation (Figure 2B). Western blot analysis showed that TGFβ_1_ stimulation in MRC5 cells results in increased phosphorylation of SMAD3, but also increased active, dephosphorylated β-catenin (Figure 3). Stimulation of MRC5 cells with Wnt antagonists SFRP1 (Figure 3A) or FRZB (Figure 3B) reduces the active fraction of β-catenin. Both SFRP1 and FRZB inhibit the TGFβ_1_-induced increase of active β-catenin, but do not influence the TGFβ_1_-induced phosphorylation levels of SMAD3, positioning Wnt signaling activity downstream of the active TGFβ signal in lung fibroblasts. Epithelial-mesenchymal transition (EMT) may also contribute to fibrosis. We therefore studied the effect of recombinant SFRP1 or FRZB and TGFβ_1_ stimulation on alveolar epithelial cells (A549). Recombinant SFRP1 does not alter baseline *E-cadherin* levels, nor the TGFβ_1_-induced downregulation of *E-cadherin* in A549 cells. However, SFRP1 significantly reduced TGFβ1-induced upregulation of *coll1α1* (Figure 4A). FRZB did not alter TGFβ_1_-induced alterations in *E-cadherin* or *coll1α1* expression in A549 cells (Figure 4B). In contrast to our observations in lung fibroblasts, TGFβ_1_ does not increase active β-catenin in alveolar epithelial cells (Figure 5).

### Absence of *Sfrp1* or *Frzb* does not affect fibrotic responses in the bleomycin-induced lung fibrosis model

**Figure 6:**
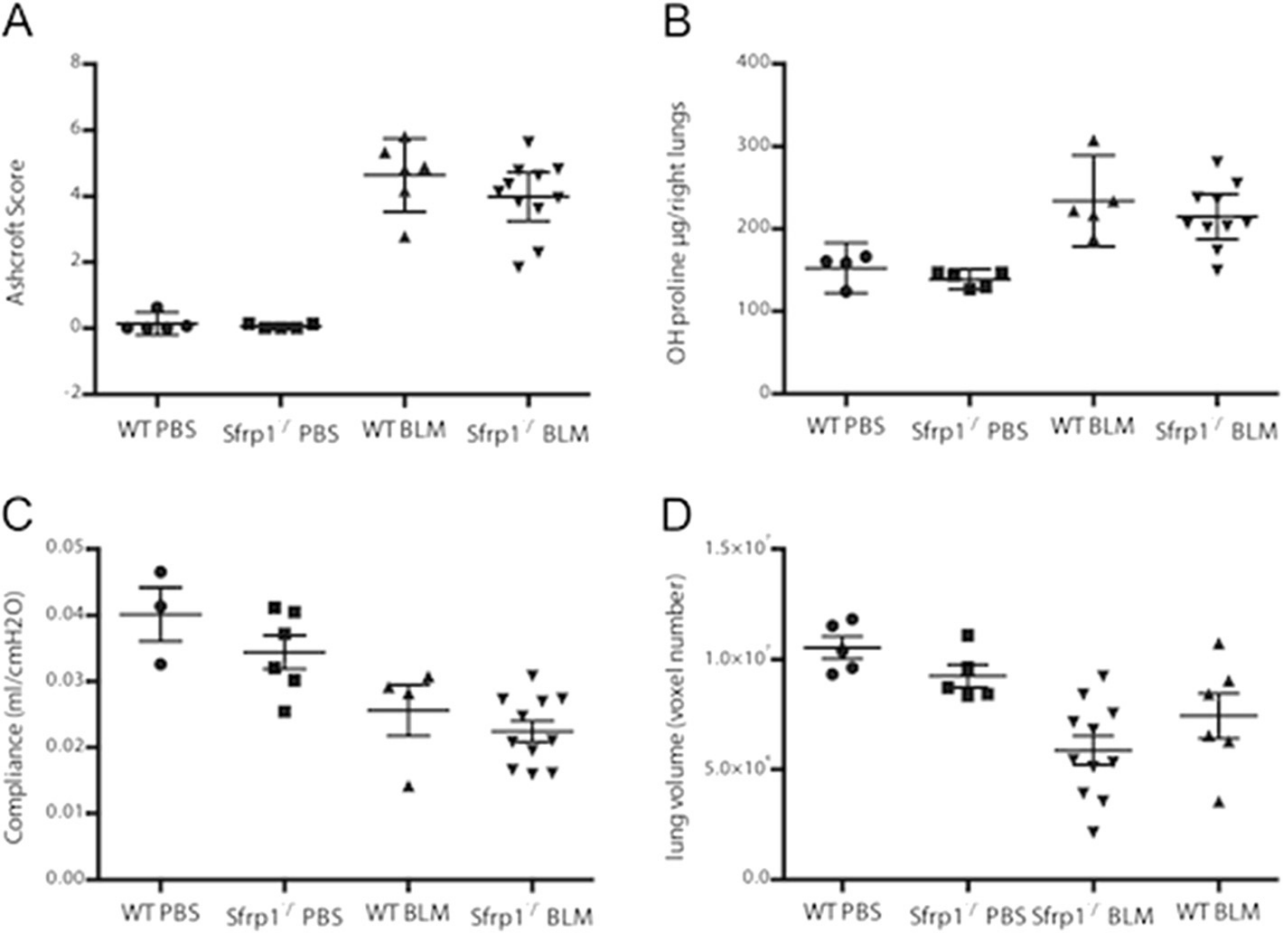
**Bleomycin-induced lung fibrosis in *Sfrp1***^***−/− ***^**mice. (A)** Ashcroft score 4 weeks after PBS or bleomycin instillation, from WT mice (PBS n = 5, BLM n = 6) and *Sfrp1*^*−/−*^ mice (PBS n = 5, BLM n = 11). **(B)** Hydroxyproline content of left lungs 4 weeks after PBS or bleomycin instillation from WT mice (PBS n = 4, BLM n = 5) and *Sfrp1*^*−/−*^ mice (PBS n = 5, BLM n = 10). **(C)** Pulmonary compliance 4 weeks after PBS or bleomycin instillation of WT mice (PBS n = 3, BLM n = 4) and *Sfrp1*^*−/−*^ mice (PBS n = 6, BLM n = 11). **(D)** End-expiratory volume (EEV) 4 weeks after PBS or bleomycin instillation, quantified by μCT imaging in WT mice (PBS n = 5, BLM n = 8) and *Sfrp1*^*−/−*^ mice (PBS n = 5, BLM n = 11).

**Figure 7:**
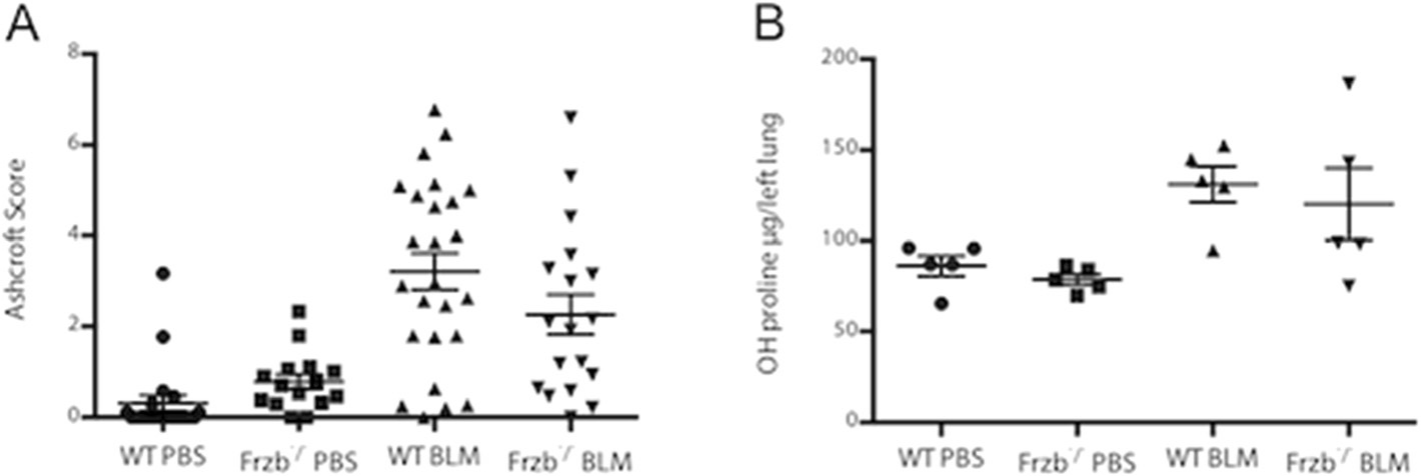
**Bleomycin-induced lung fibrosis in *Frzb1***^***−/− ***^**mice. (A)** Ashcroft score 4 weeks after PBS or bleomycin instillation, from WT mice (PBS n = 21, BLM n = 25) and *Frzb*^*−/−*^ mice (PBS n = 15, BLM n = 18). **(B)** Hydroxyproline content of left lungs 4 weeks after PBS or bleomycin instillation, from WT mice (PBS n = 5, BLM n = 5) and *Frzb*^*−/−*^ mice (PBS n = 5, BLM n = 5).

Based on these *in vitro* observations and the expression profile during bleomycin-induced lung fibrosis, we further studied the role of endogenous SFRP1 and FRZB using the respective knockout mice compared to wild-type (WT) littermates. The severity of pulmonary fibrosis, induced by intratracheal bleomycin instillation, was identical in *Sfrp1*^*−/−*^ mice compared to WT mice (Figure 6). Histopathological Ashcroft score (Figure 6A), collagen content (Figure 6B), pulmonary compliance (Figure 6C), and end-expiratory volume (EEV), quantified by μCT imaging (Figure 6D) were comparable between *Sfrp1*^*−/−*^ mice and WT mice. Likewise, upon instillation of bleomycin, *Frzb*^*−/−*^ mice have similar fibrotic responses when compared to WT mice. *Frzb*^*−/−*^ mice have an increased Ashcroft score (Figure 7A) and increased lung collagen content by bleomycin (Figure 7B), comparable to WT controls.

## Discussion

Wnt signaling is crucial in embryonic lung development. Growing evidence highlights a role for increased functional Wnt signaling in fibrogenesis. Wnt pathway activity is tightly regulated at the extracellular level. SFRPs are extracellular WNT antagonists that share sequence similarity with the Fz receptors and bind WNT ligands, preventing their interaction with their dedicated receptor complex. Extracellular Wnt antagonists are molecules of therapeutic interest to control Wnt signaling activity and alter its oncogenic, pro-invasive, and fibrogenic properties. In this work, we investigated the modulatory roles of SFRP1 and FRZB in TGFβ-stimulated pulmonary fibroblasts and epithelial cells. Next, we studied fibrotic responses in bleomycin-induced lung fibrosis in mice lacking one SFRP family member, using *Sfrp1*^*−/−*^ and *Frzb*^*−/−*^ mice.

In the bleomycin lung fibrosis model, we confirm the presence of increased active canonical Wnt signal by immunohistochemistry for β-catenin. Gene expression analysis of whole lung lysates showed concurrent upregulation of the Wnt antagonists *Sfrp1* and *Frzb* in the bleomycin model. The related antagonists *Sfrp1* and *Sfrp2* are 100-fold more expressed than *Frzb* and *Sfrp4*. However, only *Sfrp1* and *Frzb* show dynamic changes in their expression levels upon bleomycin instillation. We therefore focused on SFRP1 and FRZB. In an attempt to localize SFRP1 or FRZB protein in the bleomycin model, we performed multiple immunohistochemistry optimization steps, but were unable to eliminate false positive background staining for both SFRPs. Western blot analysis of whole lung lysates confirmed the increased presence of SFRP1 in bleomycin-treated lungs, but no band was detected for FRZB, not even in the positive recombinant protein control, indicating technical shortcomings. We hypothesize that both SFRPs are upregulated in bleomycin-induced lung fibrosis, in an attempt to control overactive canonical Wnt signaling. We therefore studied the antifibrotic potential of both SFRP1 and FRZB in a series of *in vitro* experiments.

In pulmonary fibroblasts, we confirm that an active TGFβ_1_ signal results in downstream activation of canonical Wnt signaling, which can be inhibited by exogenous administration of SFRP1 and FRZB. *In vitro*, co-stimulation of pulmonary fibroblasts with both TGFβ_1_ and SFRP1 but not FRZB abolishes the TGFβ_1_-induced upregulation of collagen expression. These data support the notion of a modulatory role of SFRP1 and FRZB in the downstream signaling events of TGFβ in fibroblasts. The differential effect of SFRP1 and FRZB on TGFβ_1_-driven collagen expression is noteworthy as both SFRPs equally abolish the activation of β-catenin. Wnts signal both through canonical β-catenin dependent pathways, and through non-canonical pathways. SFRPs are known to be able to modulate both. In recent years, the interest in this SFRP family has grown, as increasing evidence indicates that SFRPs are not only Wnt binding proteins, but can also antagonize one another’s activity, bind to FZD, interact with other receptors or matrix molecules and interfere with BMP signaling, increasingly expanding the biological relevance of these molecules [19,22]. The pleiotropic effects of the SFRP family members might explain this disparity. Interestingly, in pulmonary epithelial cells, TGFβ induces EMT, evident by a downregulation of epithelial and upregulation of mesenchymal markers, but this effect does not alter β-catenin phosphorylation levels. Likewise, in epithelial cells, SFRP1, but not FRZB, reduces the profibrotic effects of TGFβ.

From these *in vitro* datasets, we concluded that both SFRP1 and FRZB could alter TGFβ signaling in fibroblasts. As both *Sfrp1*^*−/−*^ and *Frzb*^*−/−*^ mice were available, we decided to study the potential modulatory roles of both SFRPs in the bleomycin-induced model, using a loss-of-function approach. *Sfrp1*^*−/−*^ mice have impaired distal lung development with dilation of the alveolar ducts. This finding was shown to be non-progressive, not altering lung mechanics, and thus considered a developmental defect, not an ongoing destructive process [23]. In our study we did not find differences in base-line parameters between *Sfrp1*^*−/−*^ or *Frzb*^*−/−*^ mice and littermates, including pulmonary function tests and μCT imaging.

In our experiments, loss of neither SFRP1 nor FRZB in *Sfrp1*^*−/−*^ or *Frzb*^*−/−*^ mice alters fibrotic outcomes compared to WT mice. These findings might be explained by functional redundancy between the different SFRP subgroup members, where other SFRP family members could functionally compensate for the loss of one member. Indeed, different SFRP knockout animals have only discrete phenotypes, whereas double knockout animals have been reported with severe developmental abnormalities. As we have both *Sfrp1*^*−/−*^ and *Frzb*^*−/−*^ mice available, we have tried to setup double knockout animals. However, live births of *Sfrp1*^*−/−*^*/Frzb*^*−/−*^ double knockout mice are very rare, suggesting the existence of developmental problems that are currently under further investigation and go beyond the purpose of this study.

## Conclusion

Wnt and TGFβ signaling play a central role in fibrogenesis. In this work, we examined the role of two secreted Wnt antagonists, SFRP1 and FRZB. We show that SFRP1 counteracts the profibrotic effect of TGFβ_1_ in pulmonary cells *in vitro*. However, loss of neither SFRP1 nor FRZB alters fibrotic outcomes in the lungs *in vivo*. The lack of *in vivo* effect in the absence of specific SFRPs suggests functional redundancy within this family of Wnt antagonists.

## Methods

### Animals

Eight-week-old male *Sfrp1*^−/−^ (a gift from Jeff Rubin, NIH) and *Frzb*^*−/−*^ mice [24], both on a C57BL/6 background were used. WT littermates served as controls. Weights were not different between the strains (mean +/− standard error (SE) WT (n = 57) 24.09 +/− 0.25 g, *Sfrp1*^−/−^ (n = 19) 23.05 +/− 0.54 g, *Frzb*^*−/−*^ (n = 33) 23.79 +/− 0.29 g). Mice were genotyped by PCR using DNA obtained from tail biopsy tissues. WT and *Frzb*^*−/−*^ knockout alleles were amplified using forward primer p1 (5′-TGAACTTTGCCCGACCTCTGAG-3′) and reverse primer p2 (5′-GATCGCTCGGATCACTTGTTGG-3′) or using forward primer p3 (5′-CTGATGTCTCTGCCAGAGCGAG-3′) and reverse primer p4 (5′-TGGACGTAAACTCCTCTTCAGACC-3′), respectively. WT and *Sfrp1*^*−/−*^ knockout alleles were amplified as previously described [25]. The KU Leuven Ethical Committee for animal research approved all experiments.

### Bleomycin-induced lung fibrosis

Lung fibrosis was induced by intratracheal instillation of 0.05 U bleomycin (BLM) (Sanofi-Aventis), dissolved in 50 μL of sterile phosphate buffered saline (PBS), or PBS as a control. An incision was made in the shaved anterior neck region. Blunt dissection of the salivary glands and the pretracheal muscles along the midline exposed the trachea. The animal was placed in 70° upright position. A 0.3 mL syringe with a 30G needle was placed between two tracheal cartilaginous rings after which BLM or PBS could slowly be injected. The wound was closed with Vicryl 5.0. Pulmonary fibrosis was induced in WT, *Sfrp1*^−/−^, and *Frzb*^*−/−*^ mice (*Sfrp1*^−/−^: BLM n = 11, PBS n = 5, WT littermates: BLM n = 6, PBS n = 5; *Frzb*^*−/−*^ BLM n = 18, PBS n = 15, WT littermates: BLM n = 25, PBS n = 21). Four weeks after baseline induction, mice were scanned with a desktop *in vivo* micro-computed tomography (μCT) imager and subsequently invasive pulmonary function tests were performed using the flexiVent® SCIREQ system. Mice were sacrificed and pulmonary tissue was collected for histopathology, collagen quantification, and gene expression analysis. For the timecourse experiment, BLM was administered and mice were sacrificed 7, 14, and 21 days after induction.

### (μCT) pulmonary imaging

Mice were scanned in supine position using a desktop *in vivo* small animal μCT (SkyScan 1076, software version 3.2, Kontich, Belgium) at day 28 after induction. Images were acquired and retrospective respiratory gating applied. Images were acquired in list-mode with the following parameters: 50 kVp X-ray source voltage, 180 μA current, a composite X-ray filter of 0.5 mm aluminium, 120 ms camera exposure time per projection, 9 projections per view, 23 × 35 mm field of view, acquiring projections with 0.7° increments over a total angle of 180°, producing images with a real pixel size of 35 μm. Total scanning time was approximately 12 min, resulting in a radiation dose of 813 mGy. Tomograms were reconstructed using NRecon software (version 1.6.1.3, SkyScan). Images were analyzed using CTAn software (version 1.10.0.0, SkyScan) using an automated and validated aerated lung volume segmentation algorithm, calculating end-expiratory lung volume (EEV) (corresponding to functional residual volume (FRV)) [26].

### Pulmonary function tests

Mice were anesthetized with an intraperitoneal injection of pentobarbital (70 mg/kg) (CEVA) to suppress spontaneous breathing. After a tracheostomy, the mice were connected to the flexiVent system (SCIREQ). The computer-controlled small animal apparatus ventilated the mice quasi-sinusoidally with a tidal volume of 10 mL/kg at a frequency of 150 breaths/min and a positive end-expiratory pressure of 2 cmH_2_O to achieve a mean lung volume close to that during spontaneous breathing. On the flexiVent we performed a Snapshot perturbation. Each time before performing this perturbation, a total lung capacity perturbation (TLC) was carried out to normalize the lungs. The data from the TLC perturbation were not used. The snapshot perturbation was performed until three acceptable measurements (coefficient of determination >0.95) were recorded in each individual subject, of which an average was calculated. The snapshot perturbation was imposed to measure resistance (R), compliance (C), and elastance (E) of the whole respiratory system (airways, lung, and chest wall). Only the data of the C are presented in the results [27].

### Histological analysis

After completion of invasive pulmonary function tests, mice were euthanized with pentobarbital overdose. The tracheal cannula was removed, the chest cavity was opened, and heart and lungs were removed en bloc. The left lung was collected for histopathology, inflated with 400 μL of 10% formalin/PBS via the left main bronchus and fixed in formalin for 24 h. After paraffin embedding, 5 μm sections were cut throughout the whole lung. Five sections, with 1 mm interval, were stained with hematoxylin-eosin (H & E) or Masson-Trichrome. The semi-quantitative Ashcroft score was used to score pulmonary fibrosis [28]. In short, upon 100x magnification, each successive field was given a score ranging from 0 (normal lung) to 8 (total fibrous obliteration of the field). All scores from five sections were averaged.

### Immunohistochemistry

For immunohistochemistry, sections were quenched with 3% H_2_O_2_/H_2_O. Antigen retrieval was performed in a 10 mM sodium citrate buffer and sections were preincubated with donkey serum (10% in PBS). Sections were incubated overnight at 4°C with primary antibody against β-catenin (1:200 dilution; Santa Cruz). Negative control studies were performed with species-specific IgG (Jackson ImmunoResearch). Secondary antibodies were horseradish peroxidase-conjugated antibodies (1:200 dilution; Jackson ImmunoResearch).

### Hydroxyproline assay

In the respective experiments, right lungs were collected for hydroxyproline quantification and stored at −80°C for later analysis. Hydroxyproline quantification was performed as described [29]. Lungs were hydrolyzed for 3 h in 6 M HCl at 120°C. After cooling down for 15 min, pH was neutralized (pH 6 to 7) using NaOH. Samples were diluted 1/20 in sterile H_2_0. Free hydroxyproline was oxidized with Chloramine-T for 20 min after which the oxidation reaction was stopped using 70% perchloric acid. Ehrlich’s reagent was added and samples heated for 20 min in a 60° water bath. After cooling down for 5 min, absorbance was measured at 570 nm and concentrations were calculated based on a standard curve.

### Cell culture

A549 lung adenocarcinoma cells were cultured in Dulbecco’s Modified Eagle Medium (DMEM)/Glutamax (4.5 g/L of glucose) (Invitrogen) supplemented with 10% fetal bovine serum (FBS) (Gibco), antibiotic-antimycotic solution (100 units/mL penicillin, 100 μg/mL streptomycin, and 0.25 μg/mL amphotericin B; Invitrogen), and sodium pyruvate (Gibco). MRC5 cells were cultured in Minimal Essential Medium Eagle (EMEM) supplemented with 10% FBS, minimal essential amino acids, antibiotic-antimycotic solution, and L-glutamine. Cells were allowed to attach for 24 h, after which they reached 80% confluency and were serum starved for another 24 h. Cells were stimulated with 0 to 5 ng/mL TGFβ_1_ (R & D Systems), 0 to 100 ng/mL of human recombinant SFRP1 of FRZB (R & D Systems) for 24 h for gene expression analysis.

### RNA isolation and quantitative reverse transcription-polymerase chain reaction

Total RNA was extracted from lung homogenates and cell lysates using NucleoSpin RNA isolation kit (Machery Nagel) and reverse-transcribed using First strand cDNA synthesis kit (Fermentas). Gene expression levels were quantified using Taqman Assays-on-Demand (Applied Biosystems). Expression was normalized to hypoxanthine-guanine phosphoribosyltransferase (*Hprt*) for *in vivo* experiments, and to glyceraldehyde 3-phosphate dehydrogenase (*Gapdh*) for *in vitro* experiments, and subsequently normalized to the control condition using the comparative cycle threshold method (ΔΔCT).

### Protein extraction and western blotting

Total cell lysates were homogenized in 300 μL Cell Extraction Buffer (Invitrogen) supplemented with 5% Proteinase Inhibitor Cocktail (Sigma Aldrich) and 1 mM phenylmethylsulfonyl fluoride (PMSF) (Sigma Aldrich). A total of 5 (MRC5 cells) μg or 7.5 (A549 cells) μg of proteins were loaded onto a 4-12% Bis-Tris gel (Invitrogen). Electrophoresis was carried out in running buffer (NuPAGE MES SDS running buffer (20×); Invitrogen) at 150 V for 70 min. Proteins were transferred onto a polyvinylidene difluoride membrane using semi-dry transfer (Bio-Rad) for 70 min. Non-specific binding sites were blocked for 75 min with 5% skimmed milk powder in TBS-Tween (TBST). Blots were probed with primary antibodies overnight at 4°C. The antibodies against active β-catenin (#05-665, Millipore) and total β-catenin (#610154, BD Biosciences) were used at dilutions 1/1,000 and 1/2,000, respectively in 5% skimmed milk powder in TBST. The antibodies against phosphorylated SMAD3 (#9520, Cell signaling) and total SMAD3 (#9523, Cell signaling) were used at dilution 1/1,000 in 5% Bovine Serum Albumin (BSA) in TBST. Stripped blots were probed with Actin (#A2066, Sigma Aldrich) in TBST 5% BSA at 1/8,000 as loading control. Next day, the membrane was incubated with a horseradish peroxidase (HRP)-conjugated goat anti-mouse secondary antibody (dilution 1/10,000 in 5% skimmed milk powder in TBST) (Jackson ImmunoResearch) for active and total β-catenin and anti-rabbit IgG conjugated with HRP abtibody (dilution 1/50,000 in blocking buffer) (Jackson ImmunoResearch) for phosphorylated and total SMAD3 and Actin, for 1 h at room temperature. All washes were performed with TBST (3 × 10 min).

### Statistical analysis

Data were analyzed using GraphPad Prism 6.0 (Graphpad). Data were analyzed by one-way ANOVA with Holm-Sidak *post-hoc* test for multiple group comparisons or two-way ANOVA where appropriate. *P* values <0.05 were considered significant.

## Abbreviations

A549: Alveolar epithelial cells; BLM: Bleomycin; BSA: Bovine serum albumin; C: Compliance; cAMP: Cyclic adenosine monophosphate; CBP: CREB binding protein; CREB: cAMP response element binding protein; Dkk: Dickkopf; DMEM: Dulbecco’s Modified Eagle Medium; E: Elastance; EEV: End-expiratory lung volume; EMEM: Minimal Essential Medium Eagle; EMT: Epithelial-mesenchymal transition; FBS: Fetal bovine serum; FRV: Functional residual volume; FRZB: Frizzled-related protein; Fz: Frizzled; GAPDH: Glyceraldehyde 3-phosphate dehydrogenase; H & E: Hematoxylin-eosin; HPRT: Hypoxanthine-guanine phosphoribosyltransferase; HRP: Horseradish peroxidase; IPF: Idiopathic pulmonary fibrosis; JNK: Jun-amino-terminal kinase; LRP: Low-density lipoprotein receptor-related protein; MRC5: Lung fibroblast cell line; PBS: Phosphate buffered saline; PKA: Protein kinase A; PMSF: Phenylmethylsulfonyl fluoride; R: Resistance; SFRP1: Secreted Frizzled related protein-1; SSc: Systemic sclerosis; TBST: TBS-Tween; TGFβ1: Transforming growth factor β_1_; TLC: Total lung capacity; WISP1: WNT1-inducible signaling protein 1; WNT: Wingless-type like; WT: Wild-type; μCT: Micro-computed tomography.

## Competing interests

The authors declare that they have no competing interests.

## Authors’ contributions

EDL carried out the animal and *in vitro* experiments and drafted the manuscript. CA-L carried out the immunoassays. VDV and JA performed the pulmonary function tests with subsequent data analysis. FPL participated in the design of the study. RJL participated in the design of the study and performed the statistical analysis. All authors read and approved the final manuscript.
